# MYB Transcription Factors in Chinese Pear (*Pyrus bretschneider*i Rehd.): Genome-Wide Identification, Classification, and Expression Profiling during Fruit Development

**DOI:** 10.3389/fpls.2016.00577

**Published:** 2016-04-29

**Authors:** Yunpeng Cao, Yahui Han, Dahui Li, Yi Lin, Yongping Cai

**Affiliations:** School of Life Sciences, Anhui Agricultural UniversityHefei, China

**Keywords:** pear, MYB transcription factor, gene family, expression, lignin synthesis

## Abstract

The *MYB* family is one of the largest families of transcription factors in plants. Although, some *MYB*s were reported to play roles in secondary metabolism, no comprehensive study of the *MYB* family in Chinese pear (*Pyrus bretschneideri* Rehd.) has been reported. In the present study, we performed genome-wide analysis of *MYB* genes in Chinese pear, designated as *PbMYBs*, including analyses of their phylogenic relationships, structures, chromosomal locations, promoter regions, GO annotations, and collinearity. A total of 129 *PbMYB* genes were identified in the pear genome and were divided into 31 subgroups based on phylogenetic analysis. These *PbMYBs* were unevenly distributed among 16 chromosomes (total of 17 chromosomes). The occurrence of gene duplication events indicated that whole-genome duplication and segmental duplication likely played key roles in expansion of the *PbMYB* gene family. Ka/Ks analysis suggested that the duplicated *PbMYBs* mainly experienced purifying selection with restrictive functional divergence after the duplication events. Interspecies microsynteny analysis revealed maximum orthology between pear and peach, followed by plum and strawberry. Subsequently, the expression patterns of 20 *PbMYB* genes that may be involved in lignin biosynthesis according to their phylogenetic relationships were examined throughout fruit development. Among the 20 genes examined, *PbMYB25* and *PbMYB52* exhibited expression patterns consistent with the typical variations in the lignin content previously reported. Moreover, sub-cellular localization analysis revealed that two proteins PbMYB25 and PbMYB52 were localized to the nucleus. All together, *PbMYB25* and *PbMYB52* were inferred to be candidate genes involved in the regulation of lignin biosynthesis during the development of pear fruit. This study provides useful information for further functional analysis of the MYB gene family in pear.

## Introduction

Transcription factors (TFs), which usually consist of at least a DNA-binding domain, transactivation domain, nuclear localization signal and oligomerization site, are important regulators of gene transcription. The *MYB* gene family has been reported to be one of the largest TF families in the plant kingdom. Each MYB family member possesses an MYB DNA-binding domain of approximated 52 amino acids in length at its amino-terminus. The basic structure of this DNA-binding domain includes a helix-turn-helix (HTH) fold with three regularly spaced tryptophan (Trp) residues. Upon binding to DNA, the HTH structure intercalates into the major groove (Lipsick, 1996; Stracke et al., 2001). The first MYB TF reported, v-MYB, was identified in avian myeloblastosis virus (Klempnauer et al., 1982). Subsequently, three types of MYB TFs (c-MYB, A-MYB, and B-MYB) were identified in diverse vertebrates, insects, fungi, and slime molds (Lipsick, 1996; Martin and Paz-Ares, 1997; Rosinski and Atchley, 1998; Weston, 1998). C1, the first plant MYB TF identified in *Zea mays*, encodes a c-MYB-like TF involved in anthocyanin biosynthesis (Paz-Ares et al., 1987). Based on the number of adjacent MYB repeats, MYB TFs can be divided into several different classes: 1R-MYB, R2R3-MYB, 3R-MYB, and 4R-MYB (Du et al., 2012b). R2R3-MYB and 1R-MYB are the main types of TFs identified (Kranz et al., 2000; Yanhui et al., 2006). R2R3-MYB TFs are the most prevalent in plants and are thought to have evolved from the 3R-MYB gene (Jiang et al., 2004a). The *Oryza sativa* genome contains 88 *R2R3-MYB*, 62 *1R-MYB*, and 4 *3R-MYB* genes, respectively (Katiyar et al., 2012), and the *Arabidopsis thaliana* genome includes 5 *3R-MYB*s, 135 *R2R3-MYB*s, and 52 *1R-MYB*s (Stracke et al., 2001; Durbarry et al., 2005; Yanhui et al., 2006). R2R3-MYB TFs are likely involved in the regulation of plant-specific processes, including primary and secondary metabolism, developmental processes, cell apoptosis and identification and responses to biotic and abiotic stresses (Kranz et al., 2000; Haga et al., 2007; Dubos et al., 2010). For example, overexpression of *ZmMYB31* and *ZmMYB42* in *A. thaliana* has been shown to result in decreases in both *COMT* gene expression and the lignin content (Fornalé et al., 2009). *PyMYB10* plays a role in anthocyanin synthesis in red pear (Feng et al., 2010). Further, *AtMYB96* promotes salicylic acid biosynthesis through the ABA signaling pathway, thereby regulating stomatal movement, drought tolerance, and disease resistance in *Arabidopsis* (Seo et al., 2009; Seo and Park, 2010). An R2R3-type MYB is required for cold acclimation in *Arabidopsis* (Zhu et al., 2005). Ectopic expression of an apple MYB gene, *MdMYB10*, in *Arabidopsis* enhances its tolerance to osmotic stress (Gao et al., 2011). In addition, transgenic *Arabidopsis* plants with *FLP* and *AtMYB88* exhibit elevated tolerance to abiotic stress by restricting cell divisions late in the stomatal cell lineage (Xie et al., 2010).

Pear is a commercial fruit crop available worldwide. Cai et al. (2010) have reported that the stone cell content is an important factor impacting the taste of pear fruit. Pear stone cells are mainly composed of lignin. Therefore, reduction of the lignin content in pear stone cells may improve the quality of pear fruit. Previous studies have provided much information on the monomer compositions, connector types and lignin contents in pear fruit throughout its developmental stages (Cai et al., 2010; Jin et al., 2013). In addition, the functions of some MYB TFs in secondary metabolism have been characterized in pear and other fruit trees (Feng et al., 2010). However, the *MYB* genes related to lignin synthesis in pear have not yet been identified. In the present study, genome-wide analysis of the *MYB* family in Chinese pear (*Pyrus bretschneideri* Rehd.) was performed, including database searches using the *PbMYB* gene model and analyses of phylogenetic relationships, gene structures, chromosomal locations, and other structural characteristics. Furthermore, expression analysis of the *PbMYB*s by qRT-PCR resulted in the identification of two *MYB* members that may play important roles in lignin synthesis during pear fruit development. In addition, identification and analysis of the *MYB* genes related to lignin synthesis in pear will help to improve the quality of pear fruit.

## Materials and Methods

### Identification and Characteristics of the *MYB* Genes in Pear

Identification of the non-redundant *MYB* genes in the pear genome^1^ was performed using DNATOOLS software. First, the consensus protein sequences (PF00249) of the MYB hidden Markov model (HMM) were downloaded from Pfam^2^. Then, this HMM profile was used as a query to identify all MYB-containing sequences in pear by searching against the pear genome with an E-value of <1e^−3^. Subsequently, all candidate PbMYBs were verified using Pfam and SMART^3^ to confirm that they contained the core domains. Based on the sequence alignments generated by ClustalX software, all potentially redundant MYB sequences were discarded.

Analysis of exons and introns was carried out using Gene Structure Display Server 2.0^4^ (Guo et al., 2007) by comparing the coding sequences (CDS) with their corresponding gene sequences. The isoelectric point (PI) and protein molecular weight (kDa) of each PbMYB were obtained using ExPASy proteomics server^5^. MapInspect ^6^ was used to determine the locations of the *PbMYB* genes on the pear chromosomes. The MYB proteins were analyzed using MEME (Multiple Expectation Maximization for Motif Elicitation ^7^; Bailey and Elkan, 1995) to confirm the presence of the conserved motifs with the following parameters: an optimum motif width of no less than 6 and no more than 200 and a maximum number of motifs of 20. The conserved motifs were annotated by Pfam^8^ and SMART^3^.

### Sequence Conservation Analysis

The sequences of the R2 and R3 domains of 105 PbR2R3-MYBs were aligned using ClustalW^9^ to analyze their sequence features. Multiple alignment files for these domains were submitted to WebLogo^10^ (Crooks et al., 2004) using the default settings to acquire sequence logos.

### Analysis of *cis*-Acting Elements in the Promoter Regions of *PbMYB*s

To analyze the promoter regions of the *PbMYB* genes, 2,000 bp regions upstream of these genes were examined based on the positions of the genes provided in pear GigaDB database^1^. PLACE database^11^ (Higo et al., 1999) was employed to examine putative *cis*-acting regulatory DNA elements in the promoter regions of these genes.

### Phylogenetic and Collinearity Analyses

A total of 133 MYB protein sequences in *A. thaliana* were obtained from TAIR^12^. In addition, 18 protein sequences of well-known plant *R2R3-MYB* genes were collected from GenBank^13^. Based on the findings of previous studies (Riechmann et al., 2000; Jiang et al., 2004b), AtCDC5 is an R2R3-MYB; thus, we determined whether its orthologs exist in the pear genome in our phylogenetic analysis. We also included the 4RMYB and 3R-MYB proteins in this analysis. A phylogenetic dendrogram was constructed from the ClustalW-aligned MYB proteins by the neighbor-joining (NJ) method using MEGA5.2 software^14^ (Tamura et al., 2011) with the default parameter values.

In this study, to identify collinearity, the whole-genome sequences of pear were downloaded to our local server, and then Multiple Collinearity Scan toolkit (MCscanx; Wang et al., 2012) was used to detect the microsynteny relationships between each pair of chromosomes. The resulting microsynteny chains were evaluated using ColinearScan software with an E-value of <1e^−10^.

### Gene Ontology (GO) Annotation Analysis

The PbMYBs protein sequences were aligned to the NCBI non-redundant (nr) protein database by BlastP using Blast2GO software^15^ (Conesa et al., 2005) with the default parameters. After obtaining a GO annotation for each PbMYB, we plotted the GO classifications using WEGO online tool^16^ (Ye et al., 2006).

### Analysis of Orthologous Gene Pairs

The position of each *PbMYB* was marked using a Perl script. Orthologs of the *MYB* genes in pear, strawberry (*Fragaria vesca*), yang mei (*Prunus mume*), and peach (*Prunus persica*) were identified using OrthoMCL^17^ (Li et al., 2003). These *MYB* orthologs were confirmed by performing a reciprocal BLAST search. Then, we used Circos^18^ (Krzywinski et al., 2009) to plot the relationships of the orthologous pairs among the four species.

### Calculation of Ratios of Non-synonymous (Ka) to Synonymous (Ks) Nucleotide Substitutions

DnaSP v5.0 software (Librado and Rozas, 2009) was used to calculate Ks and Ka substitution rates. Then, the selection modes of *PbMYB* paralogs were determined by analyzing the Ka/Ks ratios. Additionally, sliding window analysis was performed to determine the Ka/Ks ratios of all of the encoding sites of the *PbMYB* paralogs.

### RNA Extraction and qRT-PCR Analysis

To examine *PbMYB* gene expression, pear (*P. bretschneideri* cv. *Dangshan Su*) fruit samples were collected on 19 April (15 days), 14 May (39 days), 21 May (47 days), 30 May (55 days), 6 June (63 days), 22 June (79 days), 15 July (102 days), and 29 August (145 days) in 2015 after flowering (DAF). Three or more fruits were collected at each stage from 40-years-old pear trees grown on a farm in Dangshan, Anhui, China. Total RNA was extracted from the collected samples using Trizol reagent (Invitrogen). Then, the DNase-treated RNA was reverse transcribed using M-MLV reverse transcriptase (Invitrogen). Primers (Supplementary Table S1) were designed for real-time quantitative PCR (qRT-PCR) using Primer Express 3.0 software (Applied Biosystems). The *tubulin* gene (Wu et al., 2012) was used as an internal reference, and the primers for this gene were synthesized by Sangon Biotech, Co., Ltd. (Shanghai, country-region China). qRT-PCR was performed using an ABI7500 instrument to examine the gene expression in cDNA samples from the cross-pollinated varieties at different developmental stages. Each reaction was performed in triplicate. The relative expression levels of the *PbMYB* genes were calculated using the 2^−ΔΔCT^ method (Livak and Schmittgen, 2001). The reaction mixtures contained the following in a total volume of 20 μl: 10 μl of SYBR Premix Ex Taq II (2x), 1 μl of template cDNA, 0.5 μl of forward and reverse primers, and water. PCR amplification was carried out under the following conditions: 50°C for 2 min and 95°C for 30 s, followed by 40 cycles of 95°C for 15 s, 60°C for 20 s, and 72°C for 20 s.

### Subcellular Localization of PbMYB25 and PbMYB52

The full-length CDS of *PbMYB25* and *PbMYB52* were cloned from pear and constructed into pCambia1304 and pCambia1301 vectors (Clontech, Beijing, country-region China), which both contain a CaMV 35S promoter and *GFP* gene, resulting in fusion PbMYB-GFP genes. The primers used for gene cloning and vector construction are shown in Supplementary Table S1. The constructed pPbMYB-GFP vectors were electroporated into *Agrobacterium tumefaciens* EHA105 using a Gene Pulser Xcell (BIO-RAD, country-region USA). The suspensions were infiltrated into the leaves of *Nicotiana tabacum* using the injection method. The expressed PbMYB-GFP was observed by confocal laser scanning microscopy (CarlZeiss LSM710, Germany).

## Results

### Identification and Annotation of *PbMYB* Genes from Chinese White Pear

To identify the MYB family genes in the pear genome, the *MYB* HMM profile (Pfam: 00249) was used as query in a BlastP search against the pear genome database. This search resulted in the identification of 138 candidate PbMYB proteins in pear, and their sequences were examined to further verify whether they contained MYB-DNA binding domains using Pfam database^8^. Four PbMYB candidates were discarded because they lacked these binding domains. Notably, *PbMYB38* (Chr15: 9477433–9479211) and *PbMYB39* (Chr15: 9296590–9298368) encode proteins that are the same length and have the same PI and molecular weight (Supplementary Table S2). Further analysis showed that *PbMYB80* (17385133–17386109) and *PbMYB83* (17513104–17514080) on chromosome 10, *PbMYB103* (8444013–8447010), *PbMYB104* (8385864–8388861) and *PbMYB105* (7592807–7596377) on chromosome 3, and *PbMYB131* (24626034–24629526) and *PbMYB133* (19686471–19689983) on chromosome 17 were also identical. Thus, the five overlapping genes were removed. As a result, a total of 129 non-redundant *MYB* genes, including 22 R1-*MYB*s, 105 R2R3-*MYB*s, and two R1R2R3-*MYB*s, some of which might be pseudogenes, were identified in pear based on the presence of one, two or three MYB-DNA binding domain repeats (Supplementary Table S2).

### Analyses of Phylogenetic Relationships and Exon–Intron Structures of *PbMYB* Gene Family Members

**FIGURE 1:**
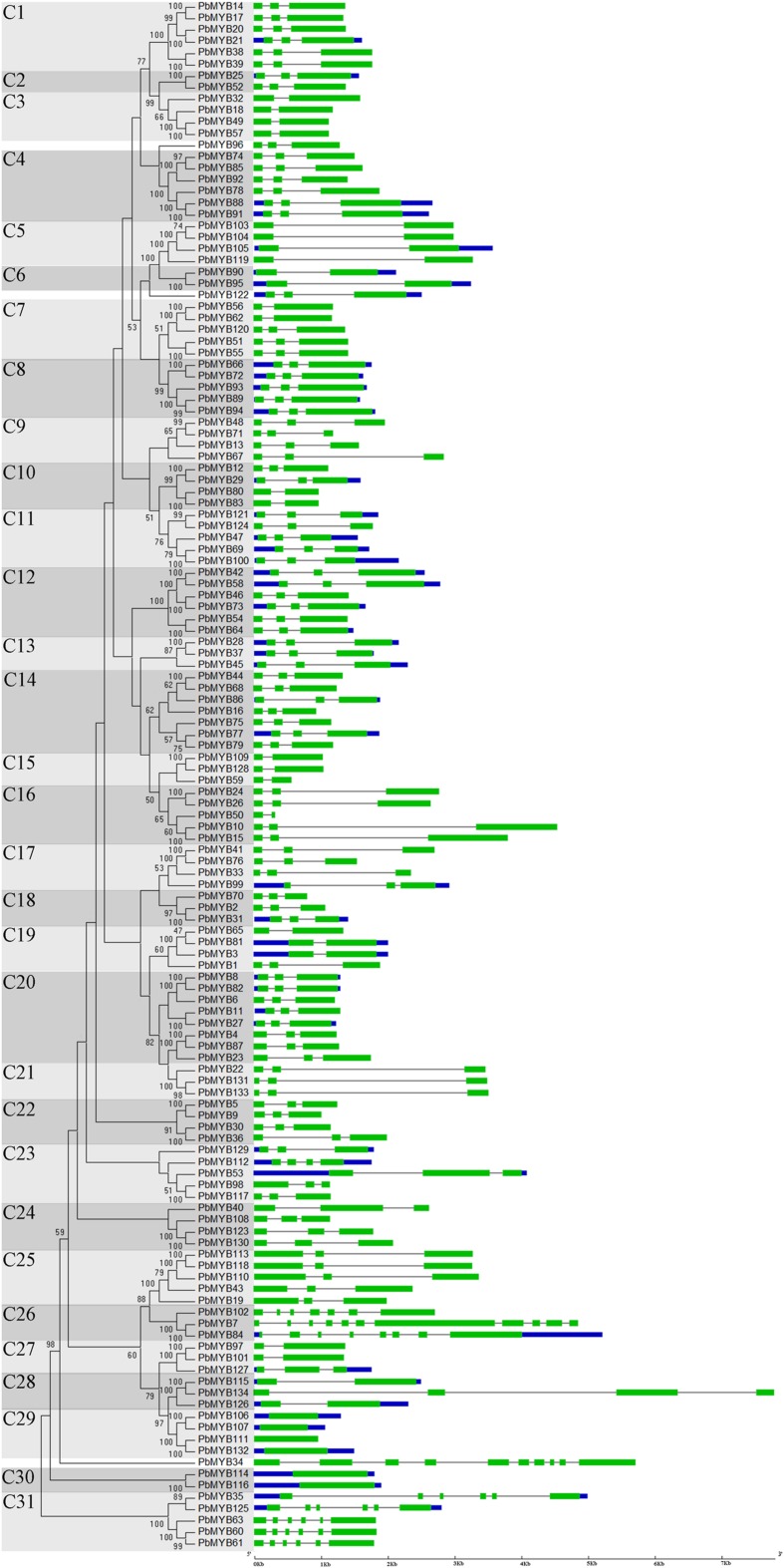
**Phylogenetic analysis and exon–intron structures of pear MYB proteins.** Complete alignments of all PbMYB proteins were used to construct a phylogenetic tree. The bootstrap values are indicated on the nodes of the branches. The green boxes, gray lines, and blue lines in the exon–intron structure diagram represent exons, introns, and UTRs, respectively. The scale on the bottom is provided as a reference.

We performed phylogenetic reconstruction of all of the *PbMYB* genes using the NJ method (**Figure 1**). A phylogenetic tree was created, in which the *PbMYB* genes were categorized into 31 major groups (C1-C31) with supported bootstrap values. The results of subsequent analysis of their exon–intron structures were consistent with those of phylogenetic analysis. Most genes clustered in the same group exhibited similar exon–intron structures, particularly with regard to the number of introns, such as C1, C2, C3, etc. (**Figure 1**). However, a few exceptional cases were observed. For example, the members of C19 contained different numbers of introns. In addition, their intron lengths were variable, ranging from several 10s of 1000s to approximately 25,000 nucleotides. These results demonstrate the presence of highly conserved structures within the *PbMYB* subfamily and high sequence diversity among the different *PbMYB* groups.

**FIGURE 2:**
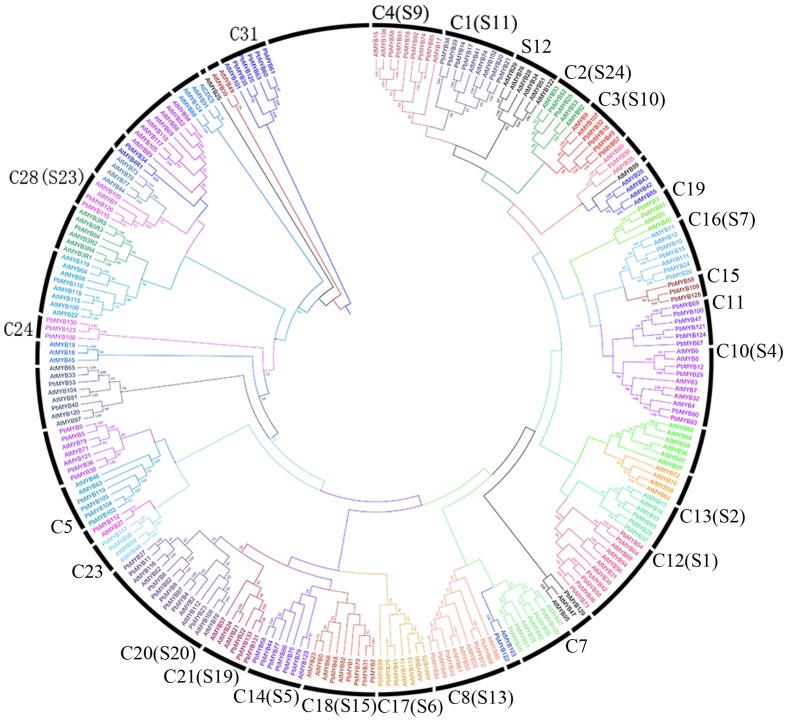
**Phylogenetic relationships of MYB proteins between pear and *Arabidopsis*.** Complete alignments of 238 MYB proteins between pear and *Arabidopsis* were performed to construct a phylogenetic tree. The bootstrap values are indicated on the nodes of the branches.

A composite evolutionary tree was generated to evaluate the evolutionary relationships of the *MYB* genes between pear and *Arabidopsis*. These members could be divided into 41 groups with bootstrap support of > 50%. Each subfamily is outlined in a different color in **Figure 2**. Most of the *PbMYB* genes were clustered with their counterparts in *Arabidopsis* with high bootstrap support in the phylogenetic tree (**Figure 2**). In previous studies (Stracke et al., 2001; Dubos et al., 2010), 126 *AtMYBs* from *Arabidopsis* were grouped into 25 subfamilies, and the defined clades are labeled in our composite evolutionary tree (**Figure 2**). Most large subfamilies in our study (C1, C2, C3, C26, etc.) are supported by the previous studies with high bootstrap support. Nevertheless, some small subfamilies (C23, C24, C15 etc.) were not retrieved from the NJ trees (Stracke et al., 2001; Dubos et al., 2010). For example, the subfamily C24 only included *PbMYBs* and not *AtMYBs*, indicating that some changes occurred among *MYB*s of different species during the evolutionary process.

### Analysis of PbMYB Domain Characteristics

**FIGURE 3:**
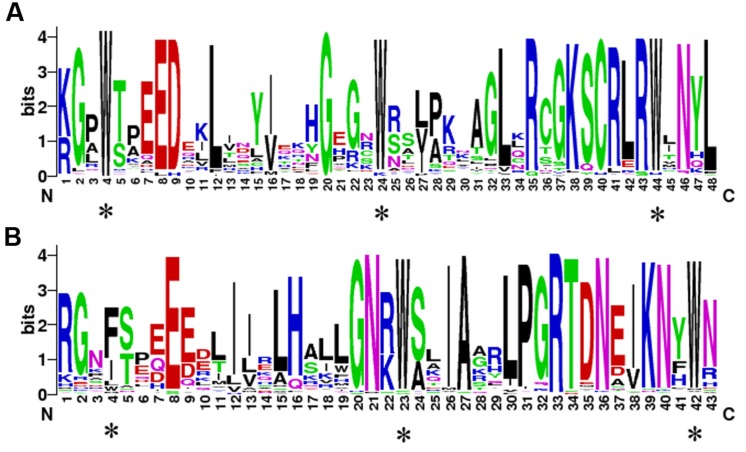
**The sequence logos of the R2 **(A)** and R3 **(B)** MYB repeats.** These logos were based on multiple alignment analysis of 105 typical PbR2R3-MYBs. The bit score indicates the information content for each position in the sequence. The asterisks indicate the typical conserved Trp residues in the MYB domain.

To demonstrate conservation at particular positions, WebLogo was used to generate sequence logos. As shown in **Figure 3**, the conserved amino acids among the PbMYB domains are very similar to those of *Z. mays, A. thaliana, Cucumis sativus, Vitis vinifera, Populus trichocarpa, Glycine max*, and *O. sativa* (Du et al., 2012b). These results indicate that many conserved amino acids are present in the R2 and R3 repeats of PbMYBs, particularly the characteristic Trp (**Figures 3A,B**). Three conserved Trp residues were identified in the R2 repeat. However, only the second and third Trp were conserved in the R3 repeat, and the first Trp was commonly substituted with Phe or Ile. Subsequently, 20 conserved motifs were identified in the pear MYB proteins using MEME web server. The MYB DNA-binding domains were represented by motifs 1, 2, 4, and 8. As shown in **Supplementary Figure S1** and Supplementary Table S3, the majority of the PbMYB proteins contained several of motifs 1, 2, 4, and 8. For example, subfamily C31 only contained motif 8, while subfamilies C28 and C29 contained motifs 1 and 4. Most of the closely related genes had the same motif compositions, indicating that there are functional similarities between MYB proteins within the same subfamily. Furthermore, we detected some subfamily specific motifs, which might be required for subfamily specific functions, such as motif 20 in subfamily C21 and motif 8 in C31. In addition, some motifs, such as motifs 3 and 6, were present in nearly every subfamily, reflecting their importance to the functions of PbMYB proteins.

### Analyses of Chromosomal Locations and Microsynteny

Based on the starting positions of the *MYB* genes within the chromosomes, 122 *PbMYB* genes were found to be unevenly distributed among 16 pear chromosomes (**Supplementary Figure S2**). Another seven genes (*PbMYB26, PbMYB49, PbMYB51, PbMYB66, PbMYB102, PbMYB109*, and *PbMYB116*) could not be conclusively mapped to any chromosome. Chromosomes 9 and 14 contained the largest numbers of *PbMYB* genes (14), followed by chromosome 15 (11), and the lowest numbers of these genes were found on chromosomes 7 and 15 (2). High densities of *PbMYB*s were observed in several specific regions, including the tops of chromosomes 10 and 15, the tops and bottoms of chromosomes 6 and 9, and the bottoms of chromosomes 8, 11, 14, and 17; in contrast, *PbMYB*s were not detected in the middle parts of the chromosomes.

**FIGURE 4:**
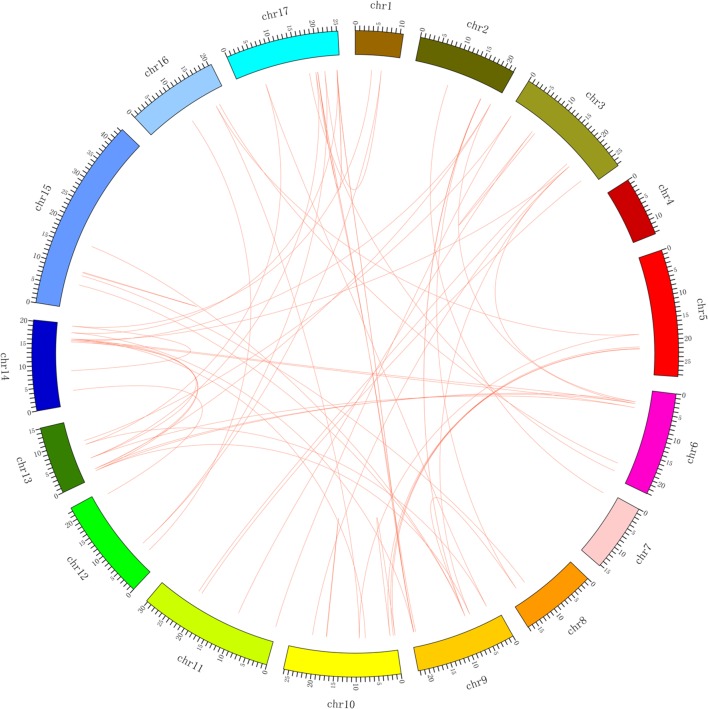
**Localization and synteny of the *MYB* genes in the pear genome.** The *MYB* genes in pear (*PbMYB*) were mapped to different chromosomes. The chromosome number is indicated on the outside. The numbers along the chromosome boxes represent sequence lengths in megabases. Gene pairs with a syntenic relationship are joined by a line.

We also investigated segmental duplication events. A total of 70 collinear gene pairs (**Figure 4**; Supplementary Table S4) were found in the pear genome, which might have resulted from whole-genome duplication in pear millions of years ago (Li et al., 2014). Among them, *PbMYB26* on scaffold 1015.0 was collinear with *PbMYB24*, and *PbMYB51* on scaffold 570.0 was collinear with *PbMYB55*, etc. The collinearity relationships of the *MYB* genes in the pear chromosomes are presented in **Figure 4** and Supplementary Table S4. Furthermore, two duplicated pairs of genes, *PbMYB50/PbMYB59* and *PbMYB113/PbMYB118*, were located on the same chromosome, and the other 68 pairs of genes were located on different chromosomes or scaffolds. Their locations within the chromosomes indicate that tandem (2) or segmental duplication (68) has occurred in these regions (Supplementary Table S4).

To explore the selective constrains among the duplicated *MYB* genes, we calculated Ks, Ka and the Ka/Ks ratio for the 70 duplicated gene pairs (**Supplementary Figure S3**; Supplementary Table S4). Generally, a Ka/Ks ratio of <1 indicates that the gene pairs are under negative or purifying selection. Further, a Ka/Ks of >1 indicates positive selection, and a Ka/Ks of =1 suggests neutral evolution. In this study, the Ka/Ks ratios for all *PbMYB* gene pairs were <1 (**Supplementary Figure S3**; Supplementary Table S4), suggesting that the *MYB* gene pairs have mainly evolved under purifying selection in pear, with limited functional divergence after the duplication events. Compared with the other replication pairs, the Ka/Ks ratios of the three pairs *PbMYB6/PbMYB11*, *PbMYB22/PbMYB131*, and *PbMYB23/PbMYB87* were larger with values of 0.82, 0.90, and 0.95, respectively. Sliding window analysis was performed to evaluate the Ka/Ks ratios of the CDS at different sites. The results showed that the Ka/Ks ratios of some coding sites were greater than 1, indicating strong positive selection (**Supplementary Figure S4**; Supplementary Table S4). As shown in **Supplementary Figure S4**, the R2 domain has undergone purifying selection, whereas the R3 domain has been mainly affected by positive selection. These results are consistent with the results of analysis of the PbMYB domain characteristics.

### Promoter Regions of *PbMYB* Genes

The promoters of the predicted *PbMYB* genes were analyzed, including a 2000 bp region upstream of the transcription start site (ATG). Five regulatory elements, including ABRE (Narusaka et al., 2003; Yamaguchi-Shinozaki and Shinozaki, 2005), LTRE (Yamaguchi-Shinozaki and Shinozaki, 2005), DRE (Narusaka et al., 2003), BOXP (Logemann et al., 1995) and BOXL (Maeda et al., 2005), were detected by performing a search of these promoter sequences against PLACE database^19^. Surprisingly, 91% of the *PbMYB* genes were found to contain putative ABRE elements in their promoter regions (Supplementary Table S5), indicating that ABA can affect the expression levels of *PbMYB* genes. In addition, some *PbMYB* genes contained BOXP and BOXL components, suggesting that they may be involved in the regulation of lignin biosynthesis. A comparison of the distributions of the five regulatory elements (ABRE, LTRE, DRE, BOXP, and BOXL) in the promoter regions among the *PbMYBs* revealed significant differences in the *cis*-elements of the promoter sequences of the 70 duplicated *MYB* genes (**Figure 4**; Supplementary Table S4), implying that the duplicated genes may have different regulatory features.

### GO Annotation Analysis

The functions of the putative PbMYB proteins in pear were predicted by GO annotation analysis. Based on amino acid similarity, 129 PbMYB proteins were categorized into 23 functional categories (**Supplementary Figure S5**; Supplementary Table S6) of the three main ontologies, namely biological process, cellular component, and molecular function. Among these categories, cell, cell part, binding, biological regulation, cellular process, metabolic process, and pigmentation were predominant. Several genes were assigned to the “death” and “anatomical structure formation” categories. Analysis of the molecular function annotations revealed that most of the PbMYB proteins function in DNA binding, followed by chromatin binding. Further, analysis of the cellular component annotations revealed that these proteins are predominantly localized to the nucleus.

### Interspecific Microsynteny Analysis

**FIGURE 5:**
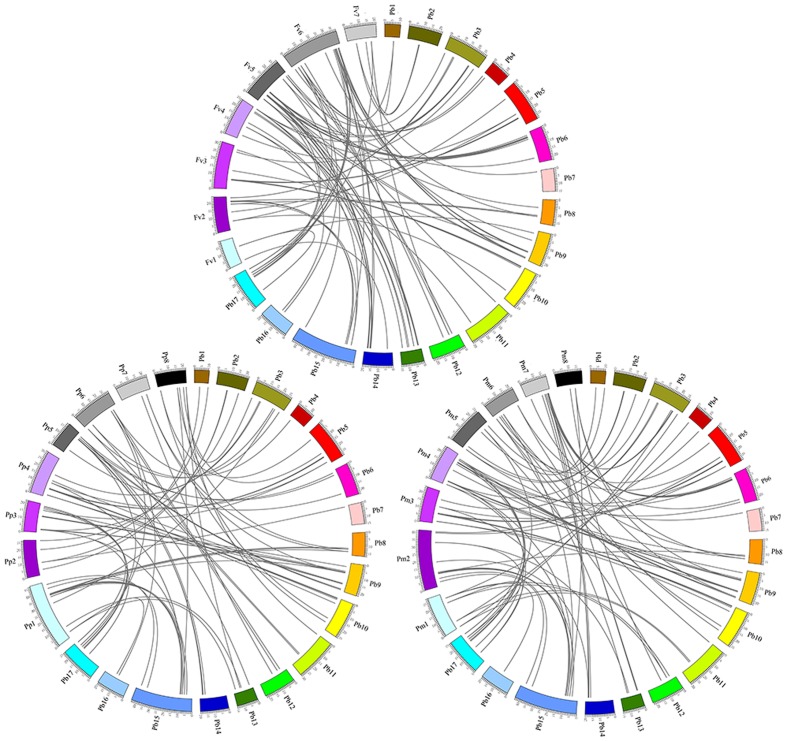
**Microsynteny of MYB regions among pear, peach and yang mei.** The chromosomes of pear, peach and yang mei, represented by the different-colored boxes, are labeled Pb, Pp, and Pm, respectively. The numbers along the chromosome boxes indicate sequence lengths in megabases. The black lines represent syntenic relationships between the MYB regions.

The orthologous *MYB* genes were identified to explore the evolutionary history of the *MYB* family in Rosaceae plants. We identified 128 orthologous gene pairs between pear and peach and 114 orthologs between pear and yang mei but only 91 orthologs between pear and strawberry (**Figure 5**; Supplementary Table S7). These findings likely reflect the closer relationship of pear with peach and yang mei (Li et al., 2014). Remarkably, some collinear gene pairs identified between pear and peach were not detected between pear and yang mei, such as *PbMYB22/ppa011751m*, *PbMYB26*/*ppa007594m* and *PbMYB43*/*ppa018123m*, indicating that these orthologous pairs formed after yang mei diverged from the common ancestor of pear and peach. Some collinear gene pairs identified between pear and peach and pear and yang mei were not identified between pear and strawberry. For example, *ppa017136m* and *Pm013279* are orthologous to *PbMYB16*, while *ppa007438m* and *Pm009892* are orthologous to *PbMYB17*, which indicates that these orthologous pairs formed after strawberry diverged from the common ancestor of pear, peach and yang mei. In addition, two or more *MYB* genes from strawberry, peach and yang mei matched one pear *MYB* gene (**Figure 5**). For example, *Pm027045*, *Pm013279* and *Pm005570* are orthologous to *PbMYB77*, *ppa007438m*, and *ppa016708m* are orthologous to *PbMYB17*, and *mrna25149* and *mrna03712* are orthologous to *PbMYB6*, suggesting that these genes are probably paralogous gene pairs.

### *MYB* Gene Expression in Pear

**FIGURE 6:**
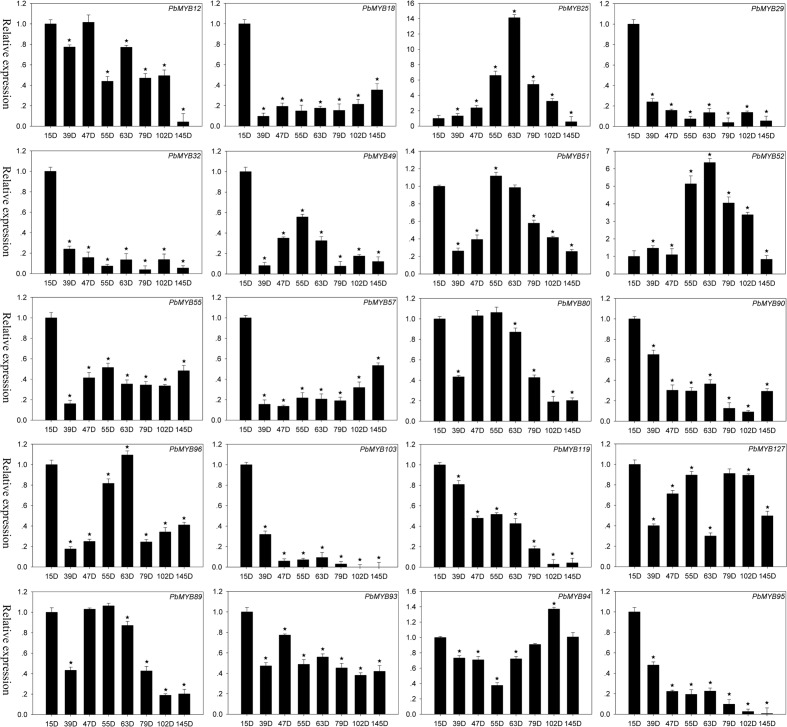
**Expression patterns of *PbMYB* genes during eight developmental stages of pear fruit at 15, 39, 47, 55, 63, 79, 102, and 145 days after flowering (DAF).** The expression profile data were obtained using qRT-PCR, and the relative expression levels were log_2_ transformed. *Significant difference compared with the expression level at 15 DAF (*p* < 0.05).

Increasing evidence indicates that MYB TFs play key roles in the regulation of plant metabolism. For example, *AtMYB32*, *AtMYB4*, *AmMYB330*, *EgMYB2*, *PttMYB21a*, etc. are involved in the regulation of lignin synthesis in plants. To identify additional *MYB* genes that participate in the regulation of lignin synthesis in pear, we generated a composite evolutionary tree that included many *MYB*s in other plants that take part in the regulation of lignin synthesis and all *PbMYB*s using the NJ method (**Supplementary Figure S6**). According to the phylogenetic relationships, we identified 20 PbMYBs (chosen from lignin-related gene subgroups, **Supplementary Figure S6**) that might participate in lignin synthesis in pear fruit. The qRT-PCR results showed that these genes exhibited diverse expression patterns at 15, 39, 47, 55, 63, 79, 102, and 145 DAF (**Figure 6**). The expression of 12 genes (*PbMYB18, PbMYB25, PbMYB29, PbMYB32, PbMYB49, PbMYB55, PbMYB57, PbMYB90, PbMYB93, PbMYB95, PbMYB103, PbMYB119*, and *PbMYB127*) was significantly increased at 15 days after blooming. Therefore, they were inferred to be involved in lignin synthesis as upstream genes. Expression of 7 *MYB* genes (*PbMYB12, PbMYB25, PbMYB51, PbMYB52, PbMYB80, PbMYB96*, and *PbMYB89*) was significantly increased at 47, 55, or 63 DAF, with similar expression patterns as the key genes involved in regulation of the lignin synthesis pathway (Xie et al., 2013). These results suggest that these TFs might be specific activators of the lignin synthesis pathway. *PbMYB94* expression was significantly increased at 102 days after blooming, indicating that this gene is involved in not only regulating the lignin pathway but also mediating the synthesis of other materials, such as flavonoids.

We further analyzed the expression of five pairs of segmental duplicated genes at eight time points after flowering. The results revealed that two of these genes had similar expression patterns at several time points, while others exhibited variable expression patterns. For example, the expression of *PbMYB55* was significantly increased at 15 DAF, while that of *PbMYB51* was significantly increased at 55 days after blooming.

### Subcellular Localization of PbMYB25 and PbMYB52

**FIGURE 7:**
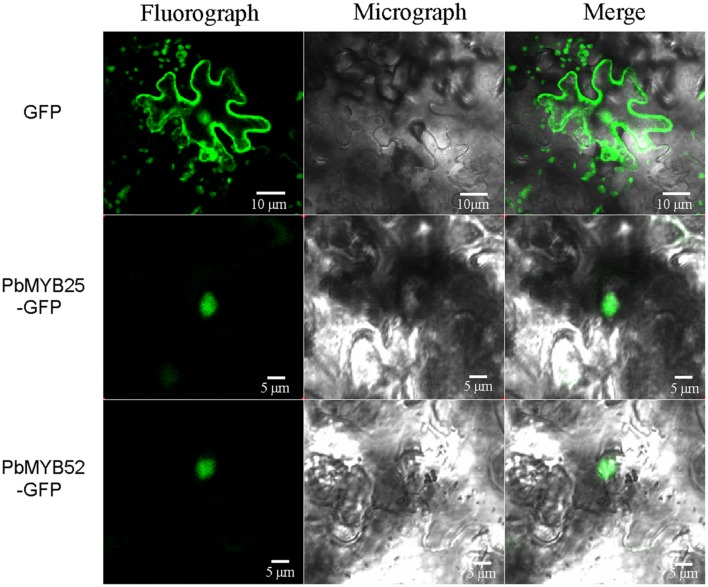
**Subcellular localization of *PbMYB25* and *PbMYB52***.

Nuclear localization of TFs is critical for their regulatory functions. Many factors influence the process of nuclear entry, such as environment stress, the cell cycle, and the developmental stage. Although, 40–60 kD proteins can pass through nuclear pores by diffusion, the movement of a protein through a nuclear pore is a proactive process. The entry process requires one or more nuclear localization signals. To examine the subcellular localization of PbMYB25 and PbMYB52, pPbMYB-GFP expression vectors were constructed and transformed into *N. tabacum*. As shown in **Figure 7**, green fluorescence signals from the expressed fusion PbMYB25-GFP and PbMYB52-GFP genes were specifically distributed within the nuclei. In contrast, green fluorescence from the expressed GFP alone was observed throughout the whole cell, demonstrating a constitutive expression pattern.

## Discussion

*MYB* genes have important functions in all eukaryotes. Systematic and comprehensive reports of whole-genome analyses of MYB family proteins have been published for *Arabidopsis* (Katiyar et al., 2012), *O. sativa* (Katiyar et al., 2012), *Z. mays* (Du et al., 2012a), *V. vinifera* (Matus et al., 2008), *Eucalyptus grandis* (Soler et al., 2015), and *Setaria italica* (Muthamilarasan et al., 2014). The pear (*P. bretschneideri* Rehd.) Genome Sequence Project was completed in 2012 (Wu et al., 2013); thus, the pear genome sequence could serve as a useful tool for genome-wide analyses of the *PbMYB* gene family. To date, no comprehensive analysis of the *PbMYB* gene family has been reported, and the functions of most *PbMYB*s are unknown. In this study, we comprehensively analyzed the *PbMYB* family, including analyses of phylogeny, gene structures, promoter regions, gene duplication events, GO annotations, chromosomal locations, sequence features, and expression profiles.

The number of *PbMYB* genes in pear (129) is higher than that in *Brachypodium* (98) (Matus et al., 2008; Muthamilarasan et al., 2014) and lower than those in *Z. mays* (132) (Du et al., 2012a), *Malus pumila Mill.* (229) (Cao et al., 2013), *O. sativa* (155) (Katiyar et al., 2012) and *Setaria italic* (209) (Muthamilarasan et al., 2014), indicating that *MYB* genes in different plants have expanded to differing degrees. Gene duplication has played a very important role in the expansion of gene families (Kent et al., 2003; Cannon et al., 2004). In this study, segmental and tandem duplications were found in a majority of the *PbMYB* genes. A total of 70 duplication events were identified in the 129 *PbMYB*s, most (68) of which involved segmental duplication, and several (2) of which involved tandem duplication. The whole-genome duplication and genome replication that occurred 5 billion years ago in Rosaceae species increased the number of chromosomes from 9 to 17 (Velasco et al., 2010), which may have resulted in the large *MYB* TF family in pear. In addition, seven *PbMYBs* could not be mapped to any chromosome, which might be due to the quality of the pear genome sequence or a high level of heterozygosity. The Ka/Ks ratios of 70 clear *PbMYB* replications indicated that the *PbMYB* gene family has generally undergone purifying selection, indicative of highly conserved evolution. However, parts of the CDS of many *PbMYBs* have undergone positive selection, indicating that new gene functions might have been acquired.

Interspecific microsynteny analysis showed that the numbers of orthologous genes between pear and peach (128) and between pear and yang mei (114) were more than that between pear and strawberry (91). These results are consistent with a previous study reporting that the divergence of peach, yang mei, and pear occurred after divergence of strawberry from the common ancestor of peach, yang mei, and pear (Wu et al., 2013; Li et al., 2015). Our results provide a novel resource for studying the evolution of *MYB* families among different species.

Examination of the phylogenetic relationships of the *MYB*s between pear and *Arabidopsis* showed that most of the clades contained different numbers of AtMYB and PbMYB proteins, suggesting that the two species display conserved evolution. To date, five *3R-MYB* genes and two *4R-MYB* genes have been identified in *Arabidopsis* (Hou et al., 2014). However, the identified *3R-MYB* genes from pear were not clustered with the *3R-MYB*s from *A. thaliana* in the phylogenetic tree, indicating that these genes are not conserved between pear and *A. thaliana* and that their functions may not be conserved in these two species. No *4R-MYB* genes were identified in pear. Moreover, *PbMYB*s and *AtMYB*s were not equally represented within the given subfamilies. For example, subfamily C24 did not contain any *AtMYB*s. These results suggest that species-specific *MYB* genes were either acquired in pear or lost in *Arabidopsis* lineages after divergence from the most recent common ancestor.

Notably, genes within the same group generally share similar exon–intron patterns, including intron numbers and distributions (Du et al., 2012b). In addition, the modal lengths of the first two exons are highly conserved (Matus et al., 2008). This finding is consistent with the results of our phylogenetic analysis and support their validity. However, in our study, the same subgroup of *PbMYB* genes consistently displayed a certain degree of divergent intron–exon organization. Similar results have been observed in apple (Cao et al., 2013), indicating that the evolution modes of MYB genes might be similar for those within the same family. During the process of evolution, certain genes in plants may be lost (Du et al., 2012b). The members of the *AtMYB* subfamily S12 have been reported to be involved in glucosinolate synthesis; however, no *MYB* genes in grape, apple or rice have been found to cluster with them (Yanhui et al., 2006; Matus et al., 2008; Cao et al., 2013). In our study, no *PbMYB*s clustered with members of subfamily S12 of *A. thaliana*, which may be due to the evolution of a specific type of gene duplication for defense against herbivores.

In general, the N-termini of MYB TFs contain a conserved DNA-binding domain (Dubos et al., 2010). This domain includes 1∼4 incomplete repeat units, each of which is composed of three helices. Normally, the second and third helices (R2 and R3) form an HLH structure for binding to DNA (Kranz et al., 1998; Li et al., 2012). A total of 105 PbR2R3-MYB proteins were identified in this study. The R2 repeats generally contained three highly conserved Trp residues, while the first Trp residue of the R3 repeats was variable. Substitution at the first Trp residue may be responsible for the recognition of novel target genes and/or may lead to loss of DNA-binding activity against target genes (Ogata et al., 1994). The N-terminal domain is relatively conserved, while the C-terminal domain has higher mutability (Stracke et al., 2001; Jiang et al., 2004a; Dubos et al., 2010; Du et al., 2012b). In this study, the MEME results revealed that the proteins PbMYB15, PbMYB89, PbMYB93, and PbMYB94 contained the conserved motif 18, which had one or more C-terminal domains. Conserved *MYB* TFs may participate in complex physiological processes via their conserved N-terminal domains and their transcriptional activation- or repression-specific C-terminal domains. For example, some *PbMYBs* were might be involved in the regulation of lignin biosynthesis in this study.

The elucidation of gene expression patterns can provide important clues regarding gene functions. Recent studies have indicated that *MYB* genes are expressed in skin, bud, and fruit of pear (Feng et al., 2010; Xie et al., 2013). However, the role of *PbMYBs* in lignin biosynthesis has remained unclear. In this study, we identified 20 *MYB*s that might participate in the lignin metabolic pathway by constructing a phylogenetic tree from known *MYB*s. The expression levels of *PbMYB25* and *PbMYB52* were consistent with the lignin contents in the pear fruit during different stages of development; their expression increased from the early to middle stages and decreased during the mature stage (Cai et al., 2010; Jin et al., 2013). Therefore, *PbMYB25* and *PbMYB52* should be considered as important candidate genes participating in the regulation of lignin synthesis. Furthermore, the subcellular localization results suggest that *PbMYB25* and *PbMYB52* are localized to the nucleus, indicating that they have nuclear functions.

## Conclusion

Genome-wide analysis, including analyses of phylogenetic relationships, gene structures, motifs and expression of *PbMYB*s, was carried out in the present study. A total of 129 non-redundant *MYB* family members were identified, including 22 *R1 MYBs*, 105 *R2R3 MYBs*, and 2 *R1R2R3 MYBs*. These *PbMYBs* were divided into 31 subfamilies, as supported by organization of the conserved domains and phylogeny. They were unevenly distributed among 16 chromosomes (total of 17 chromosomes) in pear, with the occurrence of gene duplication. Collinearity analysis indicated that gene duplication events contributed to expansion of the *MYB* family in pear. Analysis of the promoter sequences demonstrated that transcriptional regulation of the *MYB* gene family is variable among different species. Furthermore, the expression patterns, as well as the nuclear localization of both the PbMYB25 and PbMYB52 proteins, demonstrated that *PbMYB25* and *PbMYB52* might participate in the regulation of lignin synthesis in the pear fruit. The identification and analysis of these two MYB genes will help to improve the quality of pear fruit in the future. These results provide information that may facilitate further functional analyses of *PbMYB* genes to elucidate their biological roles in pear.

## Author Contributions

Conceived and designed the experiments: YuC, YH, YL, YoC. Performed the experiments: YuC, YH. Analyzed the data: YuC, YH. Wrote the paper: YuC, YH. Provided guidance on the whole study: YuC, YH, YL, and DL.

## Conflict of Interest Statement

The authors declare that the research was conducted in the absence of any commercial or financial relationships that could be construed as a potential conflict of interest.

## Footnotes

1 http://gigadb.org/dataset/100083

2 http://pfam.sanger.ac.uk/family/PF00249

3 http://smart.embl-heidelberg.de/

4 http://gsds.cbi.pku.edu.cn/

5 http://www.expasy.org/tools/

6 http://www.plantbreeding.wur.nl/uk/software_mapinspect.html

7 http://meme.sdsc.edu/meme4_3_0/intro.html

8 http://pfam.xfam.org/

9 http://www.clustal.org/

10 http://weblogo.berkeley.edu/logo.cgi

11 http://www.dna.affrc.go.jp/PLACE/signalscan.html

12 http://www.arabidopsis.org/

13 http://www.ncbi.nlm.nih.gov/

14 http://www.megasoftware.net/index.html

15 http://www.blast2go.de/

16 http://wego.genomics.org.cn/cgi-bin/wego/index.pl

17 http://orthomcl.org/orthomcl/

18 http://circos.ca/

19 http://www.dna.affrc.go.jp/PLACE/

## Supplementary Material

FIGURE S1 **Distribution of 20 putative conserved motifs in PbMYB proteins.** The detailed information of each motif including length and conserved amino acid sequence was shown in Supplementary Table S3.

FIGURE S2 **Locations of all *PbMYB* genes on the chromosomes of pear.** The scale on the left was in megabases. The numbers at the top of each bar represent different chromosomes. The gene names on the left side of each chromosome correspond to the approximate locations of each *MYB* gene.

FIGURE S3 **Sliding window plots of duplicated *PbMYB* genes.** The window size was 150 bp, and the step size was 9 bp.

FIGURE S4 **Sliding window plots of duplicated *MYB* genes in pear.** The gray blocks, from dark to light, represent the positions of R3, R2 domain. The window size is 150 bp, and the step size is 9 bp.

FIGURE S5 **Gene ontology classification of the *PbMYB* genes.** The y-axis on the right side indicates the number of genes in a category. The y-axis on the left side indicates the percentage of a specific category of genes in the main category.

FIGURE S6 **Neighbour-Joining (NJ) tree (1000 bootstraps) built using MEGA5.2 with MYB proteins of pear and other plants**.
