# Supplementary figures and images for: MYB Transcription Factors in Chinese Pear (Pyrus bretschneideri Rehd.): Genome-Wide Identification, Classification, and Expression Profiling during Fruit Development
Source: Front Plant Sci. 2016 Apr 29;7:577. doi: 10.3389/fpls.2016.00577 (PMC4850919; doi:10.3389/fpls.2016.00577)

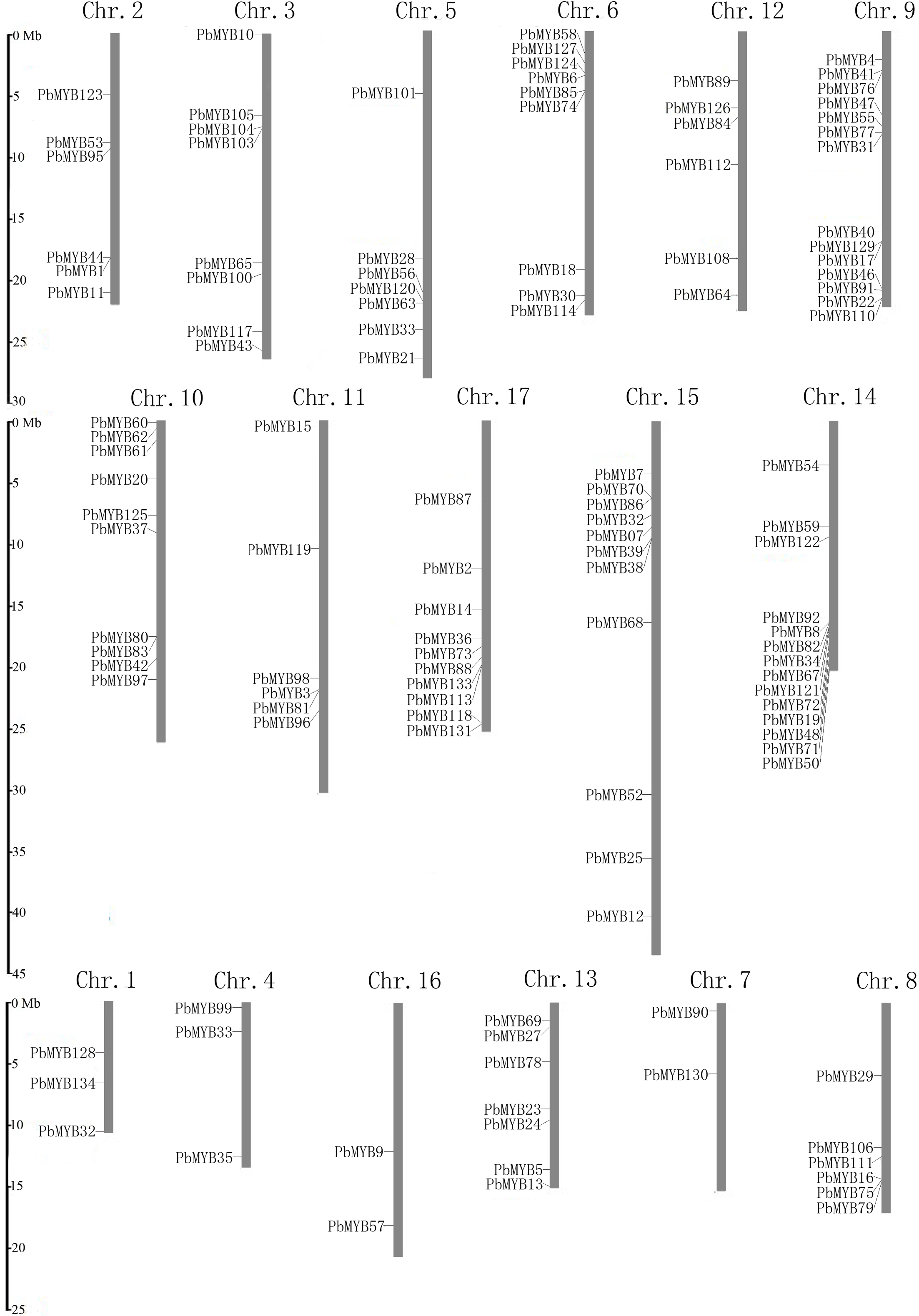

Supplement: FIGURE S2 — Locations of all PbMYB genes on the chromosomes of pear. The scale on the left was in megabases. The numbers at the top of each bar represent different chromosomes. The gene names on the left side of each chromosome correspond to the approximate locations of each MYB gene. [file Image_2.TIF]

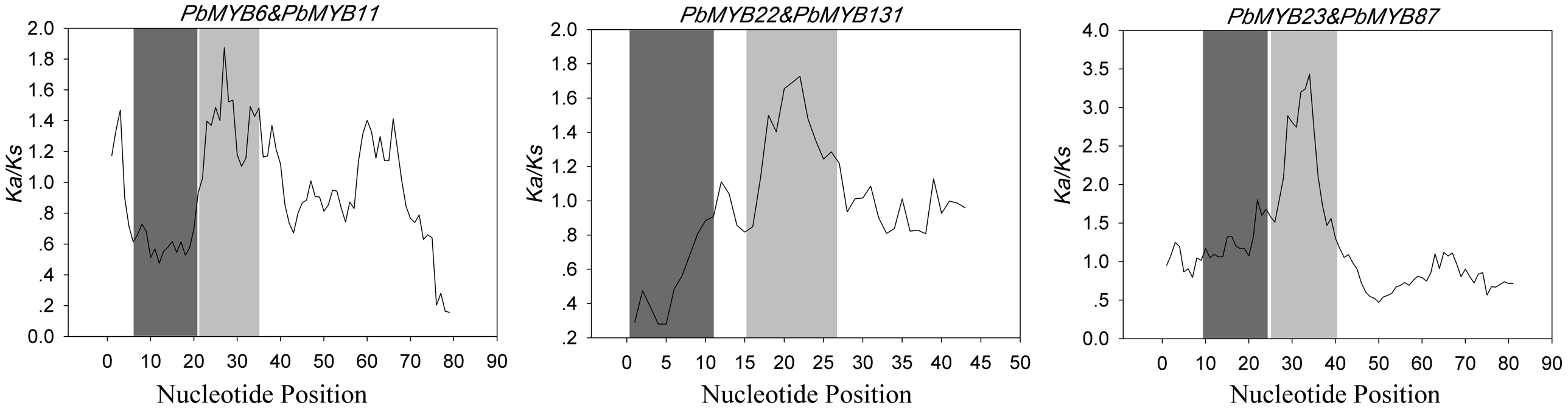

Supplement: FIGURE S4 — Sliding window plots of duplicated MYB genes in pear. The gray blocks, from dark to light, represent the positions of R3, R2 domain. The window size is 150 bp, and the step size is 9 bp. [file Image_4.TIF]

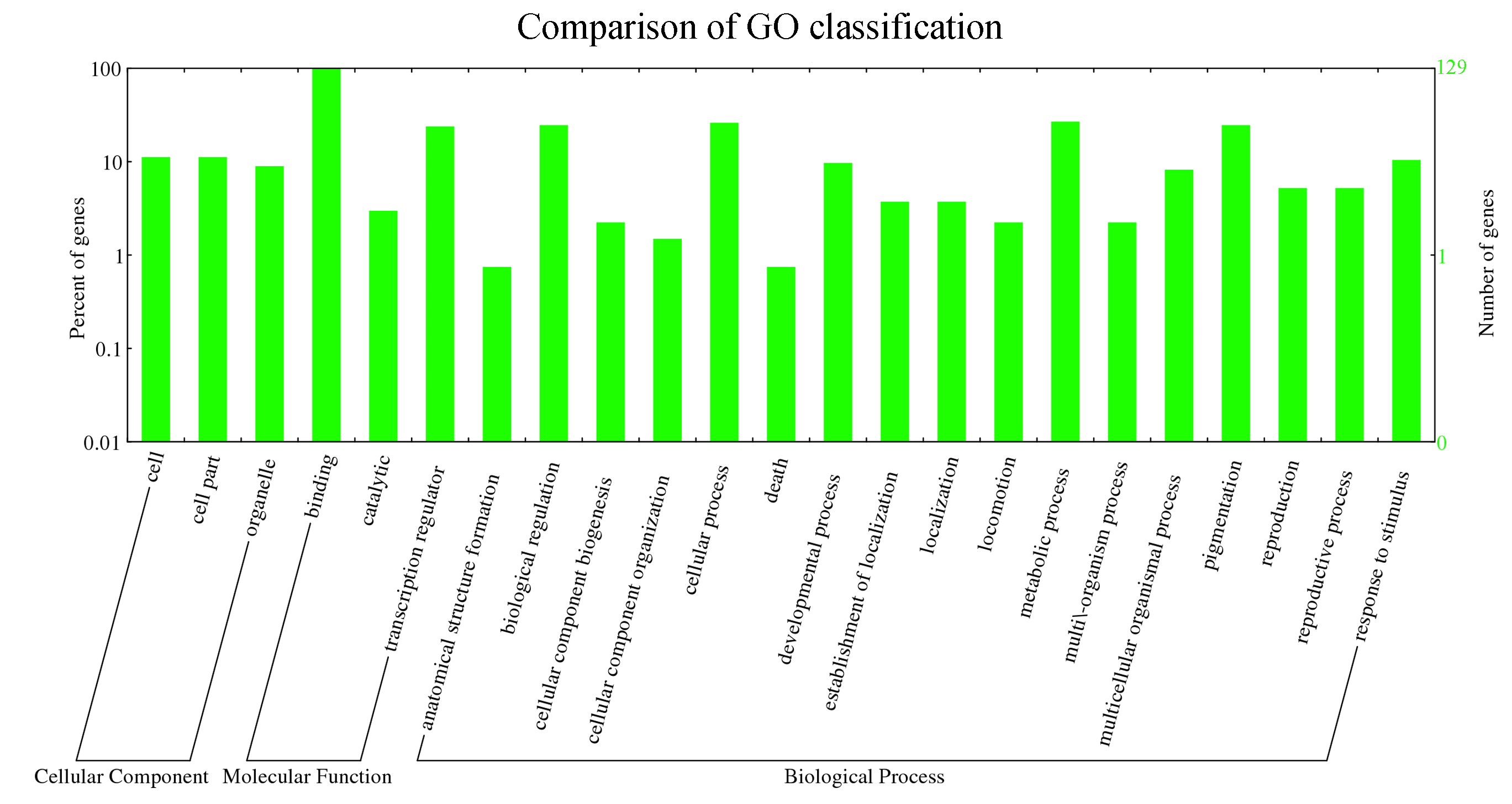

Supplement: FIGURE S5 — Gene ontology classification of the PbMYB genes. The y-axis on the right side indicates the number of genes in a category. The y-axis on the left side indicates the percentage of a specific category of genes in the main category. [file Image_5.TIF]

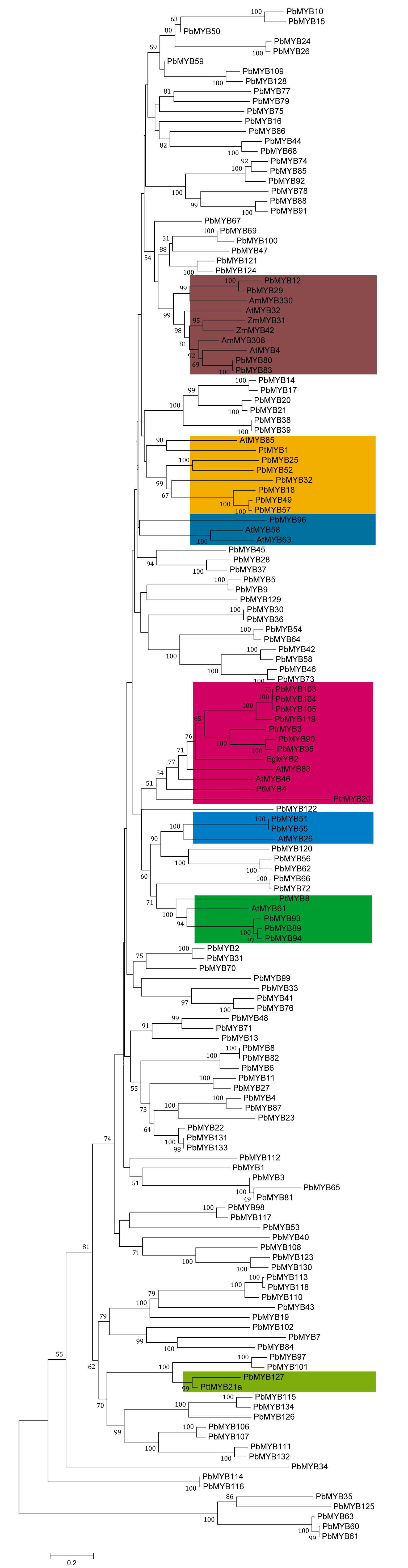

Supplement: FIGURE S6 — Neighbour-Joining (NJ) tree (1000 bootstraps) built using MEGA5.2 with MYB proteins of pear and other plants. [file Image_6.TIF]
